# Compendium of Children’s Diseases

**Published:** 1875-04-01

**Authors:** 


COMPENDIUM OF CHILDREN'S DISEASES, by Dr. Johann Steiner, Professor of the Diseases of Children in the University of Prague, etc. Translated from the second German edition, by Lawson Tait, F. C. R. S. New York: D. Appleton & Co. For sale by Jansen, McClurg & Co., Chicago. 400 pp.

The greater importance given to the study of the diseases of children in Germany, is largely due to the teachings of Henoch, Vogel, Steiner, and Korrmann. The first has enriched the literature of this subject by his excellent translations of West; the others by the compendiums which bear their names. A couple of years or so ago, the Appletons published Dr. Raphael's translation of Vogel's work, and now follow it by this reprint of an English translation of Steiner.

Prof. Steiner's position as physician for fifteen years to the immense Francis Joseph Hospital, has given him rare opportunities for the study of the diseases of infancy and childhood, the results of which he has embodied in this compact and systematic treatise of about four hundred pages. The work is divided into nine sections, which treat, respectively, of the investigation of children's diseases in general; the diseases of the nervous, respiratory, circulatory, and lymphatic systems; the diseases of the digestive, urinary and sexual organs; and diseases of nutrition; zymotic and cutaneous diseases; and lastly, a circular, giving rules for the management of infants, issued by the Birmingham Children's Hospital, with which Mr. Tait is connected. Each disease is taken up in its appropriate place; its nature, symptoms, course, anatomy, causes, diagnosis, prognosis and treatment, being systematically treated in turn. Especial care has been given to the subjects of diagnosis and pathological changes; but we find very little new in the way of treatment, which has been condensed into the smallest possible space, and seems to consist mainly of oxide of zinc, baths, stimulants, and raw beef soaked in wine.

Mr. Tait has given us an easy and faithful translation, whose only fault seems to be the preservation of such words as koprostasis, marantic, necrobrosis, helminthiasis, synostrosis, etc., where simple terms would have been better. His promised notes are also few and far between; but the book, as a whole, is a valuable one, especially as regards diagnosis and pathology, filling an intermediate place between the rather prolix work of Vogel, and the exceedingly condensed compendium of Korrmann.

H.

PRACTICE FOR SALE.

A Physician's practice, good will, etc., at one of the famous Springs, South. Price, $20,000 cash, or good secured paper. The practice now amounting to upwards of $40,000 actual cash receipts yearly, and increasing. The Physician who wishes to sell, has amassed a competency, and wishes to retire. For full particulars, please address, L. A. GILBERT,

206 La Salle Street,

Chicago, Ill.

